# Detection of genome-wide polymorphisms in the AT-rich *Plasmodium falciparum *genome using a high-density microarray

**DOI:** 10.1186/1471-2164-9-398

**Published:** 2008-08-25

**Authors:** Hongying Jiang, Ming Yi, Jianbing Mu, Louie Zhang, Al Ivens, Leszek J Klimczak, Yentram Huyen, Robert M Stephens, Xin-zhuan Su

**Affiliations:** 1 Laboratory of Malaria and Vector Research, National Institute of Allergy and Infectious Diseases, National Institutes of Health, Bethesda, MD 20892, USA; 2 Advanced Technology Program, SAIC-Frederick, Inc., NCI-Frederick, Frederick, MD 21702, USA; 3 Medical School of Drexel University, 2900 Queen Lane, Philadelphia, PA 19129, USA; 4 Pathogen Microarrays Group, The Wellcome Trust Sanger Institute, Hinxton, Cambridge, CB10 1SA, UK; 5 Bioinformatics and Computational Biosciences Branch, Office of Cyber Infrastructure and Computational Biology, National Institute of Allergy and Infectious Diseases, National Institutes of Health, Bethesda, MD 20892, USA

## Abstract

**Background:**

Genetic mapping is a powerful method to identify mutations that cause drug resistance and other phenotypic changes in the human malaria parasite *Plasmodium falciparum*. For efficient mapping of a target gene, it is often necessary to genotype a large number of polymorphic markers. Currently, a community effort is underway to collect single nucleotide polymorphisms (SNP) from the parasite genome. Here we evaluate polymorphism detection accuracy of a high-density 'tiling' microarray with 2.56 million probes by comparing single feature polymorphisms (SFP) calls from the microarray with known SNP among parasite isolates.

**Results:**

We found that probe GC content, SNP position in a probe, probe coverage, and signal ratio cutoff values were important factors for accurate detection of SFP in the parasite genome. We established a set of SFP calling parameters that could predict mSFP (SFP called by multiple overlapping probes) with high accuracy (≥ 94%) and identified 121,087 mSFP genome-wide from five parasite isolates including 40,354 unique mSFP (excluding those from multi-gene families) and ~18,000 new mSFP, producing a genetic map with an average of one unique mSFP per 570 bp. Genomic copy number variation (CNV) among the parasites was also cataloged and compared.

**Conclusion:**

A large number of mSFP were discovered from the *P. falciparum *genome using a high-density microarray, most of which were in clusters of highly polymorphic genes at chromosome ends. Our method for accurate mSFP detection and the mSFP identified will greatly facilitate large-scale studies of genome variation in the *P. falciparum *parasite and provide useful resources for mapping important parasite traits.

## Background

Malaria parasites, particularly *Plasmodium falciparum*, impose heavy economic and health burdens on human population worldwide [1]. Hundreds of millions of people are infected by the parasite each year, leading to 1–2 million deaths annually. Lack of effective vaccines and emergence of drug-resistant parasites and insecticide-resistant mosquito vectors are the main reasons for the failure in controlling the parasites and the associated disease. A better understanding of the molecular mechanisms of drug resistance, the molecular basis of the host immune response, and the strategies the parasite employs to evade host immunity is critical for vaccine and drug development.

Genetic variation in parasites can contribute to drug resistance, immune evasion, and disease manifestation. Genetic mapping is one of the powerful approaches for the identification of mutations that cause drug resistance and changes in other phenotypes [2]. For efficient mapping of a target gene, it is often necessary to genotype a large number of polymorphic markers. In addition to length polymorphisms such as microsatellites and minisatellites and large-scale sequencing, genome-wide single nucleotide polymorphisms (SNP) have been identified from many organisms, including *P. falciparum*, for genotyping and mapping genes associated with different phenotypes [3-5]. High-throughput SNP typing methods have also been developed [6-11], leading to recent successful identification of candidate genes (loci) associated with various human diseases [12-20].

One of the high-throughput typing methods is array-based hybridization. In this method, labeled genomic DNA is hybridized to microarrays comprising high-density short oligonucleotides designed based on known SNP or systematically tiled along all chromosomes to detect potential polymorphisms. High-density arrays have been successfully used to detect variation in copy number [21-23] and SNP [24,25]. The human malaria parasite *P. falciparum *has a genome with extremely high AT content (> 80%) as well as numerous repetitive sequences [26], making array design and data analysis challenging. Hybridizations of *P. falciparum *genomic DNA to both Affymetrix GeneChips^® ^and slides printed with 70 mer oligonucleotides have been reported previously [27-29]. Kidgell *et al*. recently used an array with 327,782 probes to identify 23,653 single feature polymorphisms (SFP) among 14 isolates. The results from this study suggest that high-density array could be a promising tool for high-throughput detection of genome variations including SNP and copy number variations (CNV). However, calling SNP based on hybridization signals is a complex process, and many factors can affect SNP calling, including array design, GC content of a probe, the position of the SNP in a probe, hybridization conditions, and algorithms used to analyze array signals. Additionally, methods were developed to call SFP in many previous studies, but the accuracy of SFP calls were not verified with known SNP or through DNA sequencing. To investigate the influences of these factors on calling SFP in a highly AT-rich genome and to develop a reliable method for calling SFP from the *P. falciparum *genome using commercially available array platforms, we have analyzed data from a high-density 'tiling' array with ~2.5 million 25 mer probes designed at The Sanger Institute (PFSANGER GeneChips^®^) to detect genomic variations in five *P. falciparum *field isolates. Genomic DNA samples from the five parasite isolates were hybridized to the array, and signals from the parasites were compared with known SNP [4] to evaluate SNP calling accuracy under different conditions. Based on the comparison, we identified factors that could affect probe/DNA hybridization dynamics and established a set of conditions that allowed us to call SFP/SNP with ≥ 94% accuracy. We also sequenced 52 SFP calls that did not agree with known SNP and found that ~64% of the 'wrong' calls were actually due to errors in the genome sequences. Parameters that provided best SNP calling accuracy were used to identify 121,087 potential SNP, including ~18,000 new SFP that have not been reported previously.

## Results

### Basic probe statistics and quality control

The array has 2.56 million perfect-matched probes (25 mer) with 2,206,371 *P. falciparum*-specific probes (the rest of the probes were for rodent malaria parasites). Of the *P. falciparum *probes, 2,107,319 mapped uniquely to the genome and 99,052 mapped to more than one location or were not assigned to any chromosomes. Among the unique probes, 1,446,824 were in the predicted coding regions (CDS); 1,304,180 probes were within exons; 727,200 probes were intergenic; 84,622 were within introns; 58,022 probes spanned exon/intron junctions, and 32,347 probes spanned the predicted translation start sites or stop codons.

Genomic DNA from five different parasites (Additional file 1) were labeled and hybridized (2–4 replicates) to the PFSANGER GeneChip^®^. After normalization of the hybridization signals across all array chips, an average signal intensity for each probe was calculated from replicates of each parasite. The qualities of the hybridizations were evaluated using various methods including MA plots, scatter plots (data not shown), and coefficient of variance (CV) tests (Additional file 1). Good reproducibility was obtained among replicates with the majority of the probes (> 90%) having CV less than 25% (Additional file 1). Histograms of signal ratios relative to 3D7, the reference genome, showed similar data distribution among different parasite samples (Additional file 2).

### Probe coverage of known SNP

Accurate SNP calling and detection of insertions/deletions requires optimization of calling parameters. Here we evaluated potential factors that might affect SFP calling accuracy by comparing known SNP between 3D7 and four other parasites (Dd2, HB3, 7G8, and FCR3) identified in our previous study (*i.e*., NIAID SNP) [4] and hybridization signal ratios. Among the 3,836 NIAID SNP (excluding 82 that were mapped to multiple sites) identified previously, 2,651 (69%) were covered by 10,841 probes, including 1,787 covered by 5,600 probes in the predicted exons. The majority of the SNP were covered by 1–5 probes (average 4.4 probes/SNP), with a maximum coverage of 45 probes/SNP (Additional file 3). Overall, the SNP were distributed evenly across the 25 mer positions in the probe, with ~94% of probes having one SNP (Additional file 4).

### Probe GC content and hybridization intensity

**Figure 1:**
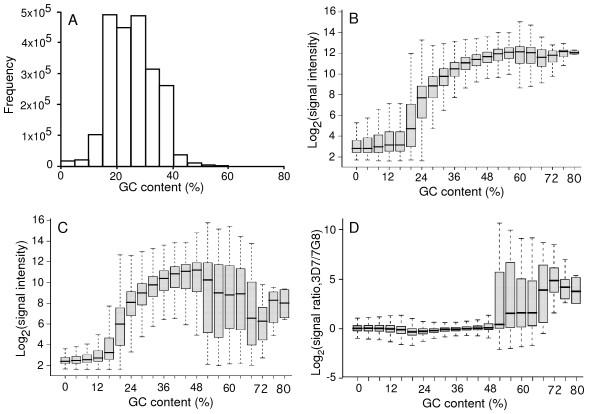
**Distribution of probes with different GC contents and the influence of GC content on signal intensity. A**. Number of probes with different GC contents. **B**. Hybridization signals from probes with different GC contents using 3D7 DNA. **C**. Hybridization signals from probes with different GC contents using DNA from 7G8. **D**. Signal ratios of 3D7 over 7G8 from probes with different GC contents. The box plots (**B-D**) showed the lowest intensity, lower quartile, median, upper quartile, and the highest intensity. Note large variations in probes with GC contents higher than 50%.

Because GC content in a probe is known to affect probe/DNA hybridization dynamics, we investigated the influence of probe GC content on hybridization signal intensity. The GC effect is likely exaggerated even more for the AT-rich genome of *P. falciparum *genome. The majority of the probes in the array have GC contents of 15% to 40% (Figure 1A). Signal intensity was similarly low for probes with GC content <16%, but for probes with GC content of 16% or higher, signal intensity increased with the increase of GC content until ~40%, when signal intensity began to plateau (Figure 1B). Signal intensity did not change much from 40% to 80% GC in 3D7; however, the intensity began to decrease and fluctuate dramatically after reaching 50% GC content in non-3D7 parasites (Figure 1C). Reduction in signal intensity in non-3D7 parasites suggested high levels of polymorphism in these probes. In the parasite genome, the first exons of the *var *gene family have a relatively high GC content and are highly variable in DNA sequence. These high-GC-content probes are therefore likely from the *var *genes. Comparison of the high-GC probes with *var *gene sequences showed that ~44% of the 5,491 probes with 50% or higher GC content were from the *var *genes. These probes likely contributed to the dramatic variation in signal ratio between parasites (Figure 1D). These results suggest that probes with GC content <16% and the *var *probes with >50% might not be reliable for the detection of SFP for genetic mapping of the *P. falciparum *traits.

### Substitution positions in a probe and hybridization dynamics

**Figure 2:**
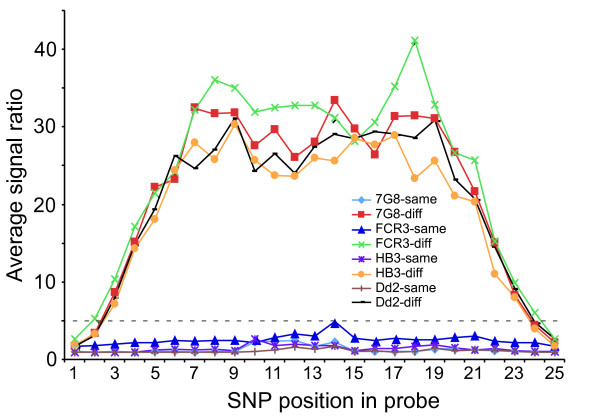
**Relationship between probe signal ratios and SNP positions.** 7G8-same indicates signals from probes with no known NIAID SNP within the probes between 3D7 and 7G8 parasites (3D7/7G8); 7G8-diff indicates probes with known differences between 3D7 and 7G8 parasites. The definitions for the rest of the parasites (FCR3, Dd2, and HB3) are the same as those for 7G8. The dashed line indicates signal cutoff ratio value of 5.0.

The position of a nucleotide substitution in a probe can also influence probe hybridization intensity. A substitution in the middle of a probe is expected to affect hybridization stability more dramatically than a change at the end positions of a probe. Comparison of average signal ratios between 3D7 and the other four parasites and SNP at known probe positions showed that substitutions at the two end positions (1 and 25) of a probe did not affect probe-target hybridization; and substitutions at position 2 and 24 had minimal effect on signal intensity (Figure 2). Signal ratios (3D7/7G8) of probes with SNP from position 3 to position 7 increased from both ends, averaging more than 10 times of the probes without polymorphism. For all positions in a probe, the average signal ratios were approximately the same (< 1.5) if there was no known polymorphism in a probe. For probes that had known SNP, the signal ratio was generally 5 or higher if two positions at each end of a probe were excluded (Figure 2). Our data showed that substitutions located at probe position 3–23 (25 mer probes) had a strong effect on hybridization intensity and should be considered for SFP detection (Figure 2).

### Estimates of correct SFP call rates

We next evaluated different signal cutoff ratios to obtain a value that produced the best SFP calling accuracy realizing that this ratio would balance false positive and false negative calling rates. We found that a signal cutoff ratio of 1.5 produced the highest overall correct call rates (≥ 90%) for Dd2, HB3, and 7G8 (Table 1). Correct call rates increased slightly after removing probes with high and low GC contents and increased further after excluding calls from single probes and calls with probe vote ratio < 75%. In contrast, correct call rates decreased with the increase of signal ratio cutoff values, likely because of the exclusion of some real SFP with relatively lower signal ratios. Even using a signal cutoff ratio of 5.0, we obtained correct call rates ≥ 85%. After correcting for wrong calls due to sequence errors (see below), we obtained correct call rates ≥ 94% (Table 1). The call rate for FCR3 could not be estimated accurately without known SNP information.

**Table 1:** Comparison of correct mSFP calling rates using different cut off values

	Overall rate			GC filtered			Probe filtered			Corrected rate		
				
Cutoff value	7G8	Dd2	HB3	7G8	Dd2	HB3	7G8	Dd2	HB3	7G8	Dd2	HB3
1.5	92.5	92.4	90.0	92.6	92.5	90.2	93.7	93.4	91.2	97.7	97.6	96.7
2.0	91.5	90.4	89.2	91.3	90.4	89.4	92.8	92.6	90.7	97.4	97.3	96.6
5.0	82.9	82.0	82.5	82.9	82.1	82.8	86.4	85.9	84.5	95.0	94.8	94.3

To obtain the best correct call rates, we compared mSFP calls using three cutoff values (1.5, 2.0, and 5.0). First we called mSFP using unique probes and probe position 3–23 (Overall rate). We repeated the calls after removing probes with GC contents < 16% and > 50% (GC filtered). We then obtained call rates after removing probes with GC content < 16% and > 50% and excluding calls with single probes and multiple probe calls with less than 75% probe votes (Probe filtered). Corrected rates were obtained after adjusting for 63.5% error rate in the wrong calls due to sequence errors, which were calculated using formula

[(100-probe filtered rate) × 0.635 + probe filtered rate].

A correct call was defined as correct calls over the sum of correct, wrong, and tie calls.

### Sequencing verification of SFP calls

Both false positive (Fp) and false negative (Fn) calls could be caused by SFP calling errors, sequencing mistakes, or problems in sequence alignment in the databases. To investigate whether the discrepancies between our SFP calls and the known SNP were from array SFP calling or sequencing/alignment errors, we sequenced 52 Fp or Fn SFP calls (positions 3–23, 1.5 cutoff ratio between 3D7 and 7G8) with different probe coverage and probe vote ratios to verify the calls. Our results showed that 33 of the 52 (63.5%) initial wrong calls were due to sequence errors in the databases, including four Fp calls that did not have polymorphism at the expected sites but had new polymorphic sites nearby, leading to the incorrect Fp calls (MAL14.5217, MAL12.3146, MAL11.3013, and PFC0210c in Table 2). Among the 19 true wrong-calls verified by sequencing, 9 were called by a single probe, 6 had mixed probes calls, 3 had two one-sided probe calls, and 1 had three one-sided probe calls. If we excluded calls from single probes and mixed probe calls having a probe vote ratio <75% (for example, one probe suggested a SFP, but three others suggested no SFP), we would have had only four calls that were incorrect (7.7% of the 52). In other words, 92% (48/52) of the calls would have been correct if we had excluded single probe calls and calls with a probe call vote ratio of <75%. If we apply these corrections, we obtain a corrected overall SFP call rate of ≥ 94% even using a conservative cutoff value of 5.0 (Table 1).

**Table 2:** DNA sequencing verification of false negative (Fn) and false positive (Fp) calls

Gene ID	Chr position	Mism alle	SFPn	3D7	7G8	Forward (5'-3')	Reversed (5'-3')
MAL2.808	chr2: 306218	T/A	Fn(0/5)	A	A	tcagtagtatcttttgtttc	atgtaaaactaccatcaaatg
**PFC0210c**	chr3: 218122	G/G	Fp(4/0)	C	C	agatgtgttctttatctaatt	aaccaagtgataagcacata
PFC0235w	chr3: 248155	A/G	Fn(0/2)	A	A	ggaaatgtatttgagaaaaac	caatgtttactatccgaatt
PFC0770c	chr3: 718081	T/T	Fp(4/0)	T	A	atggggagcaaagaatttc	tattccatgatgtattatgat
PFC1065w	chr3: 995530	C/G	Fn(1/8)	G	G	ggaaaaagaagaagatttaa	aatatatcttccgaatcatc
PFC1065w	chr3: 995640	A/G	Fn(0/8)	A	A	atagatgtatcgtgtgataa	attattacttctgtctctag
*PFE1390w*	chr5: 1154254	A/A	Fp(3/0)	T	T	cgaaaaagagaagaaaaact	tgtgttggcttcttaatatt
MAL6P1.232	chr6: 817214	T/A	Fn(0/4)	T	T	tccaaatcttctcaaagct	ggtttattcaaaacattagg
MAL7.743	chr7: 181822	C/C	Fp(5/0)	C	G	tttaatgcttccctttgctt	ataattgtgatgaagtgatg
*MAL7P1.30*	chr7: 512599	T/T	Fp(1/0)	A	A	atggtagaataattcatatgt	ttatcacacatggtttcaac
*MAL7P1.65*	chr7: 519234	T/C	Fn(0/2)	T	C	aaaacaaccgtctgatataa	taaacaataaatccaactgt
MAL7.2803	chr7: 621749	G/A	Fn(0/6)	G	G	ttttcgctcggattattaaa	gcaacatgatttttttttttc
*MAL7P1.67*	chr7: 677205	C/A	Fn(0/2)	G	T	atttaacttactggattggt	aatggacaaccaggttaaaa
MAL7P1.82	chr7: 794419	A/C	Fn(0/7)	A	A	gtgtacttcattttgtagtta	atatctacaaaaggggaatt
MAL7P1.82	chr7: 794421	C/A	Fn(0/7)	A	A	ccatgtgctttcatatatat	ccatgtaccagctcatac
PF07_0102	chr7: 922368	C/A	Fn(0/4)	C	C	aagagtattaataattccgtc	gaacagaggatgaattattt
*MAL8P1.42*	chr8: 1017925	T/A	Fn(0/1)	T	A	tccatgatatattcccaag	tattcctcatttcagggtat
MAL8.3159	chr8: 1057901	C/A	Fn(0/3)	C	C	gtacagctagttgtagtg	gagctttcttactaaagtat
*PF08_0017*	chr8: 1179041	C/T	Fn(0/1)	C	T	cggtgataataataaatacg	gaatttatagaactttccgc
PF08_0017	chr8: 169329	T/C	Fn(0/1)	T	T	ccgtctacacaataattcttt	gggtagtaaatatgaggaaa
MAL8.2086	chr8: 582828	T/C	Fn(0/4)	T	T	tgggataaacctatgtataa	tcattcaaatttacaggtcg
*PFI1300c*	chr9: 1080645	T/T	Fp(1/0)	A	A	tatgatgacaatcatattcc	ccttctatgaatagagatac
*PFI1300c*	chr9: 1080729	G/A	Fn(0/2)	T	C	tacccatatcttgatttacg	ctttggagatttgtttagat
PFI0495w	chr9: 464714	G/G	Fp(5/0)	G	A	attctcccaaaactgaaata	atatcttcgttagttatgtg
*MAL9.1104*	chr9: 548591	A/G	Fn(0/1)	A	G	tcttcttttcctttctacat	ttaaggttccttctgaatta
PFI0690c	chr9: 603205	T/A	Fn(0/2)	T	T	cgaaaaaatcctttacctt	aaagatttccccctactaaa
MAL5.878	chr9: 926274	G/A	Fn(0/1)	C	C	gttcgtcttttttttcatatg	Gaatataagacagatgttcc
PF10_0314	chr10: 1294935	C/G	Fn(3/17)	T	T	caatgtgaggaatatttatag	ggcctcattgtggttatta
*MAL10.3336*	chr10: 1334877	A/A	Fp(1/0)	A	A	tttaaacacccctcaaaaaa	aaatatcaaaaccggaaatg
MAL10.4084	chr10: 1433239	G/A	Fn(0/1)	C	C	aagaaataattggttgggct	ttctgtccaccatttttttg
*PF10_0377*	chr10: 1554669	T/A	Fn(9/11)	T	A	taaaacctgtataaccaaata	tatacaaactttacaaaactc
PF10_0094	chr10: 389999	A/C	Fn(0/2)	T	T	aaggtataccaatagatttg	gtaaatcattcaccctcat
*PF10_0138*	chr10: 556132	C/C	Fp(2/1)	C	C	taatgtgtatgtatcagcta	ggattgtaataagtatatgg
*MAL10.1222*	chr10: 564556	T/T	Fp(1/0)	T	T	gttttatgcttaggcttata	tgggaaaatataaatgaagg
*PF11_0338*	chr11: 1272493	A/A	Fp(1/0)	A	A	gaatgttaacatacaaatgta	cttcagggagaatatttattc
**MAL11.3013**	chr11: 1294419	T/T	Fp(3/0)	T	T	tcatggttcaggtataaga	ccattattttcttgagctgc
PF11_0353	chr11: 1327608	G/A	Fn(0/5)	G	G	ttataccatatgtgtacaaag	gaaatatcaaaatttcctaac
PF11_0360	chr11: 1369690	A/G	Fn(0/3)	A	A	cctattctattcaatactgt	ctgtatacatttgtttggat
PF11_0046	chr11: 151916	A/G	Fn(0/2)	A	A	acaagcatagatatcatagc	ataacatgtcctaaaggtga
*PF11_0441*	chr11: 1717528	T/A	Fn(5/15)	A	T	cagttatatacctttatcag	ataagaaaaaatatccacac
*MAL12.4052*	chr12: 1192527	T/G	Fn(1/2)	T	G	ggatattcacaatggatttt	catgtgtatcatttatacatg
*MAL12.2128*	chr12: 577914	T/T	Fp(1/0)	T	T	ctgatgaaagaatacatattg	tgaacaatatattcggaaac
**MAL12.3146**	chr12: 817466	T/T	Fp(1/0)	T	TAT	aatctaaaaaatccaagtatg	cataatgattgtatatccttt
PF13_0184	chr13: 1376386	T/C	Fn(1/9)	T	T	tattcttgaattttcgctac	tatattttatggatcatctc
MAL13.4760	chr13: 2159993	C/T	Fn(0/2)	C	C	cacaaaagtatacgtctat	ttaacagtttaggacacata
MAL13.670	chr13: 304167	C/A	Fn(0/1)	A	A	attaaataattcttcttccag	catgtcttgtatttcgtttt
MAL13P1.67	chr13: 557320	A/T	Fn(0/3)	A	A	gttcttctaacacaaataaa	tctacaggtaatatgttatc
PF13_0088	chr13: 650502	T/C	Fn(0/4)	G	G	cggcatgctcctgaagtaaa	ttatgttagagatgggtata
*PF13_0125*	chr13: 912350	T/G	Fn(2/3)	A	G	catagtactatcacctgaa	ctatggttataaccaagaaat
MAL13P1.127	chr13: 958583	A/A	Fp(2/1)	T	C	gatgaatttgttgtaacgttt	acgttaataacaatcatgtga
**MAL14.5217**	chr14: 2364467	A/A	Fp(3/0)	A	A	ggtatatcctttctacatat	aattcttttcatagggagtt
*PF14_0565*	chr14: 2428920	A/T	Fn(16/23)	T	A	atcgtcaataccttcctcg	taaacaaaatatgagcactg

Gene ID, gene ID or SNP ID in PlasmoDB; Chr position, chromosomal position of the polymorphic site; Mism Alle, mismatched alleles of our array calls and known NIAID SNP between 3D7 and 7G8; SFPn, calls not matching known SNP, either false positive (Fp) or false negative (Fn). The numbers in the parentheses are numbers of probes calling for SFP or no SFP. For example, Fp(3/0) indicates three probes called for a SFP and no probe called for no SFP, but there was no known SNP in the databases; and Fn(0/3) indicates three probes called for no SFP, but a known SNP existed (false negative); 3D7, alleles obtained from sequencing 3D7 DNA; and 7G8, alleles obtained from sequencing 7G8 DNA sequences. The gene ID in italic indicates SNP not confirmed by sequencing (true wrong calls) using 1.5 cutoff ratio and 3–23 positions in a probe; and those in **bold **had additional polymorphisms supporting the array calls. TAT in MAL12.3146 is a trinucleotide missing in 7G8. Forward and reverse are primers used in amplification and sequencing of the PCR products.

### Use of receiver operating characteristic (ROC) curves to estimate call rates

**Figure 3:**
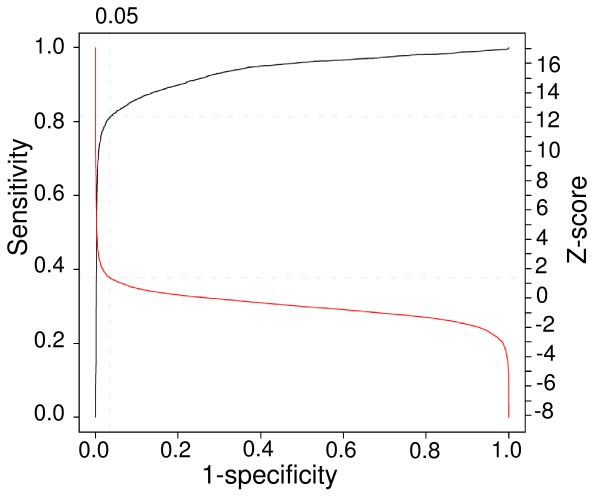
**Relationship of receiver operating characteristic (ROC) curve and Z-score values and estimates of SFP call rates.** The black line is the ROC curve, and the red line is the Z-score curve. The vertical dash line indicates false positive rate (1-specificity) of 5%, and horizon lines point to a Z-score value of 1.5 and sensitivity level (call rate) of approximately 81%, respectively. The curves were generated using data from all replicates of hybridization. SFP calls were compared with known NIAID SNP described previously (see text).

To further test the reliability of our method in calling SFP, we also used a ROC curve to evaluate SFP calling accuracy and applied local pooled error (LPE) analysis to obtain Z-scores for calling SFP [30]. LPE generates corrected Z-scores that reduce Fp, which might result when sample variance happens to be low, by using a 'pooled' variance for all the probes that show similar intensities. The ROC curve is a graphic plot of sensitivity *vs*. (1-specificty) or fraction of true positive *vs*. the fraction of Fp [31]. As shown in Figure 3, if we allowed a Fp rate of approximately 2% (1-specificity), and at a Z-score of ~1.5, we could obtain a sensitivity of call rate ~81% genome-wide for data from 7G8, Dd2, and HB3.

SFP were called using Z-scores of 1.5, 2.0, 3.0 and 4.0 and compared with SFP called using signal ratio cutoffs of 1.5, 2.0, 3.0, and 5.0. Results from cutoffs of Z-score of 3.0 and signal ratio of 3.0 had the best overall matches (~99%) and the best positive SFP call matches (~82%) for all 14 chromosomes. To minimize Fp calls (low Fp rate is important for genetic mapping) from unknown parasites that might have higher background, however, we decided to use a conservative signal ratio cutoff value of 5.0. Using this cutoff value, almost all (~98%) of the positive calls matched a positive call from a Z-score cutoff 3.0.

### Detection of genome-wide substitutions among field isolates

We used a conservative signal cutoff ratio of 5.0 and all the parameters discussed above (Additional file 5) to call SFP and obtained 121,087 mSFP genome-wide among the five parasites, including 41,700 unique mSFP from 3D7, 8,856 from 7G8, 10,068 from Dd2, 10,449 from HB3, and 5,121 from FCR3 (Table 3). Inspection of the calls revealed that the large number of 3D7 unique calls was largely from multigene families such as var, rif, and stevor. We therefore flagged mSFP from multigene families (PFB0935w, PFD0090c, MAL7P1.6, MAL7P1.58, PFI1780w, PFA0655w, PFB0105c, MAL7P1.7, MAL7P1.59, PF10_0380, PFE1600w, PF10_0012, PF10_0005) and their paralogs. Excluding mSFP from these genes removed approximately 67% of the SFP and reduced the total number of mSFP to 40,354, including 6,618 unique mSFP for 3D7, 6,855 for HB3, 2,854 for FCR3, 7,173 for Dd2, and 6,342 for 7G8 (Additional file 6). A list of SFP and mSFP in each predicted gene and genes that are highly polymorphic (genes encoding potential antigens) can be found in Additional file 7.

**Table 3:** Summary of mSFP calls for the 14 chromosomes among five parasite isolates

Isolate	Ch1	Ch2	Ch3	Ch4	Ch5	Ch6	Ch7	Ch8	Ch9	Ch10	Ch11	Ch12	Ch13	Ch14	Total
0000*	35727	54790	67238	67463	90602	85998	85713	86826	98064	190050	223105	212037	340227	231400	1869240
0001	247	615	656	896	429	506	655	524	635	765	606	1092	1486	1337	10449
0010	209	116	130	331	304	211	252	313	1529	54	390	245	393	244	5121
0011	180	41	49	244	55	137	143	269	121	173	98	165	156	76	1907
0100	349	1636	348	688	537	344	683	527	497	761	829	1224	890	755	10068
0101	248	556	73	479	279	203	336	268	193	191	178	315	164	137	3620
0110	175	165	193	491	173	267	384	199	227	506	223	488	478	222	4191
0111	296	213	235	551	941	501	406	513	389	543	378	866	597	397	6826
1000	300	278	359	563	345	467	580	598	511	1125	687	762	1287	994	8856
1001	138	241	123	427	138	401	625	315	315	621	484	342	490	327	4987
1010	179	75	70	219	55	81	117	136	49	182	275	106	209	162	1915
1011	243	288	144	533	47	593	447	438	369	363	282	348	684	316	5095
1100	123	179	166	309	87	141	343	143	78	204	177	272	424	142	2788
1101	477	271	356	860	240	1180	134	495	356	496	476	880	547	207	7975
1110	363	197	340	731	222	306	628	433	133	408	402	560	644	222	5589
1111	2507	1924	1607	3829	968	3720	4215	3149	2816	3984	3072	3948	3809	2152	41700
Total	6034	6795	4849	11151	4820	9058	10948	8320	8218	10776	8557	11613	12258	7690	121087

*Parasite isolate order is 7G8, Dd2, FCR3, and HB3. For example, '1000' indicates the numbers of unique alleles for 7G8. A '0' indicates that a parasite has the same allele as that of 3D7 (0), and '1' indicates a different allele (a mSFP). The numbers in the first row were positions with probes but no SFP were called (no polymorphism). These numbers were not counted in the total calculation. The counts were based on a signal cutoff value of 5.0. Note these calls were mSFP and were different from those defined previously, where each probe was defined as an independent SFP[28].

Some chromosomes appeared to have unusually large numbers of mSFP calls from some parasites. For example, Dd2 had 1636 unique mSFP from chromosome 2, whereas the other four parasites had fewer than 400 mSFP (Table 3). Close inspection of the calls revealed that the majority of the extra mSFP was from a deletion at one end of chromosome 2 in Dd2 (Additional files 8 and 9). Similarly, the higher numbers of mSFP from chromosome 12, 13, and 14 of HB3 were from specific regions either deleted or having highly polymorphic genes in a specific parasite (Additional file 8 and 9).

### Genome-wide mSFP distribution

**Figure 4:**
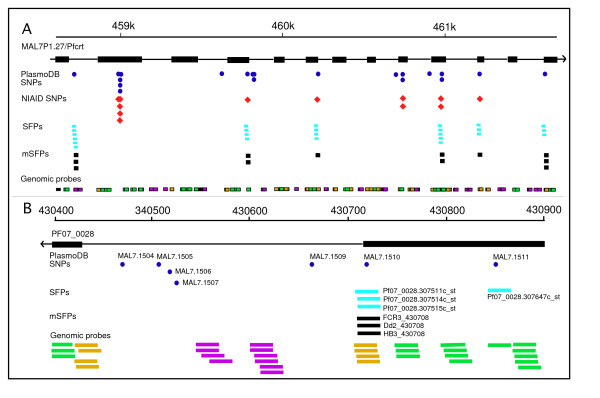
**Genome browser displays (drawn in Canvas) showing SFP, mSFP and SNP from two genomic loci on chromosome 7.A**. A genome browser window (~3 kb) showing expanded chromosome region covering *pfcrt *gene (top line) and predicted exons/introns of the p*fcrt *gene, SNP in PlasmoDB (blue circle), NIAID SNP (red diamonds), SFP from individual probe (light blue squares), mSFP (black squares) and all genomic probes covering the *pfcrt *gene. Color codes for the genomic probes are: green, probes in coding regions; purple, probes in noncoding regions; and yellow, probes spanning protein coding and noncoding regions. Note the mSFP matched well with those known SNP. **B**. An expanded region (500-bp window) from PF07_0028 showing distributions of PladmoDB SNP and array probe locations. Five of the seven PlasmoDB SNP (blue circle) in the intron were not covered by any probes. One SNP matched a mSFP call (black bars in multiple parasites), and another was covered by one probe and but was not called (filtered out because of single probe). The color codes for the genomic probes are the same as those in **A**; the labels are either SNP ID (blue circles) or probe ID (black and light blue bars).

SFP and mSFP were uploaded into the GBrowse genome browser at the ABCC website [32] for genome-wide display of the polymorphic site. Probe sequences and locations in predicted exons, introns, and intergenic regions were mapped to chromosomes. SNP in the PlasmoDB and our SFP/mSFP calls were also displayed in the browser with allele information from each parasite. As shown in the browser, the majority of our mSFP (89%) matched well with the PlasmoDB SNP (estimated for 7G8 only), including SNP in the *pfcrt *(Figure 4A). This comparison identified ~18,000 new unique mSFP (excluding those from multi-gene families) from the five parasite genomes.

We noticed that many of the PlasmoDB SNP (51.1%) were located on chromosomal regions that did not have probe coverage (Figure 4). Because the majority of the regions without probe coverage were likely in areas of AT-rich repetitive and/or noncoding sequences, the observation suggested that relatively larger numbers of SNP in the PlasmoDB could be from repetitive sequences.

We next counted mSFP in a window of 10-kb segments and plotted mSFP from each segment along the chromosomes to investigate mSFP distribution on the chromosomes from each parasite (Additional file 8). Again, these plots showed clusters of some highly polymorphic regions, mostly at chromosome ends, corresponding to *var/rif/stevor *clusters. The plots also identified some unique peaks for individual parasite, for example, a unique peak on chromosome 2 for Dd2 and HB3, respectively. These unique peaks were likely due to deleted DNA segments or reflected the unique selection and evolutionary histories in an individual parasite (Additional file 8).

### Genome-wide CNV

**Figure 5:**
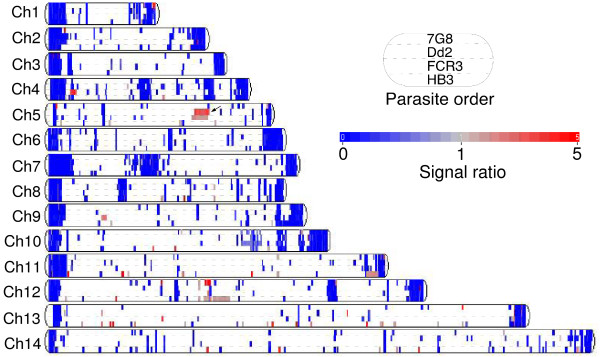
**Copy number/segmentation analyses showing amplified and highly variable or deleted regions on 14 chromosomes.** Amplified/deleted regions were displayed as a signal heat map (red, amplified; blue, deleted or highly polymorphic) from each parasite. The 14 chromosome diagrams showed amplified (red, > 1.5) or deleted/highly variable regions (blue, < 0.67) after filtering for regions 0.3 kb or larger. The dashed lines separate the four parasites in each chromosome in the order of 7G8, Dd2, FCR3, and HB3. The arrow indicates the chromosome 5 regions amplified in Dd2 and FCR3.

Genome-wide segmentation analyses showed that there were relatively few large-scale amplifications or deletions among the parasites (Figure 5). The 5 largest amplified regions were a ~28 kb on chromosome 4 of FCR3, a ~80–96 kb on chromosome 5 of Dd2 and FCR3, a ~30 kb on chromosome 9 of FCR3, a ~82.5 kb on chromosome 11 for HB3, and various sizes (~3–180 kb) in the middle of chromosome 12 for different parasites. The chromosome 5 amplified region contained a total of 20 unique genes, including 19 genes (PFE1065w-PFE1155c) amplified ~2–3 copies in FCR3 and 14 genes (PFE1095w-PFE1160w) amplified ~4–5 copies in Dd2 (Additional file 9) with a total of 13 genes shared by the two parasites. Eight of the shared genes were predicted to encode proteins related to ribosomal subunits, ATP-dependant helicase, nucleotide binding, s-adenosylmethionine-dependent methyltransferase, mitochondrial processing peptidase, G10, and multidrug resistance homolog protein, PfPgh-1. Similarly, segments of different sizes located at the middle of chromosome 12 were amplified ~7–8 copies in 7G8 (PFL1085w, PFL1125c-PFL1160c, ~67 kb), ~5 copies in Dd2 (PFL1085w, PFL1145w-PFL1150c, ~3 kb), ~3–4 copies in FCR3 (PFL1135c-PFL1160c, ~20kb), and ~2–3 copies in HB3 (PFL1085w, PFL1125w-PFL1310c, ~184kb). Only two genes (PFL1145w and PFL1150c) were amplified in all of the four parasites, one of which was a gene encoding putative ribosomal protein L24. A large region on chromosome 11 from HB3 containing 26 genes (PF11_0489 to PF11_0513) was amplified 2-3X, four of the genes were predicted to encode ring-infected erythrocyte surface antigen, antigen 332, and Ser/Thr protein kinase. The amplified region on chromosome 4 of FCR3 (~25 kb) contained genes encoding a putative reticulocyte-binding protein 1 and four hypothetical proteins (PFD0095c-PFD0115c) and was amplified at least five times. This amplified segment may play a role in the higher growth rate for this parasite, because the reticulocyte-binding protein may facilitate parasite invasion.

The majority of the regions with reduced signals (blue) were located on chromosomes ends or regions containing the *var/rif/stevor *gene clusters, reflecting the highly variable nature of these DNA regions (Figure 5). Although it is difficult to distinguish highly polymorphic regions from deletions in this haploid genome, we considered several additional restrictions to exclude potential polymorphic loci. A segment was considered not truly deleted if it contained known highly polymorphic genes such as *var/rif/stevor *[29] or if a segment had reduced signals in all four parasites (suggesting highly polymorphic genes such as genes encoding surface proteins). For segments with reduced signal ratios occurring only in one or two parasites, they were more likely to be true deletions, which could also be detected in mSFP distribution plots (Additional file 8). For example, a deletion of ~42-kb segment (PFB0070w-PFB0100c) on chromosome 2 of Dd2 and FCR3 was found to contain a gene encoding knob-associated histidine-rich protein (KAHRP). Deletion of KAHRP in Dd2 was reported previously [28,29,33]. Another likely deleted segment was a ~98-kb region on chromosome 9 of HB3 containing 19 genes (PFI1710w-PFI1800w) including the gene encoding cytoadherence linked asexual protein (CLAG) and lysophospholipase. Again, deletion of this region had been reported [34]. A list of chromosome segments and mapped genes potentially amplified or deleted/highly polymorphic, including those reported previously, can be found in Additional file 9.

## Discussion

The PFSANGER array, despite having ~2.2 million *P. falciparum *probes, was not designed specifically for SNP detection, and whether it was suitable for SNP detection was not certain. This study was initiated to investigate the possibility of using the PFSANGER array for genetic mapping and population studies. The large number of probes on the chip and their high AT content (some > 80%) require critical evaluation of factors that may affect hybridization dynamics before SFP can be reliably called. Based on comparison of mSFP calls with known SNP identified previously [4], we showed that the last two end positions in a probe had limited influence on hybridization signal and that probes with GC contents lower than 16% should be excluded for SFP calling in this genome. We also found that mSFP calls based on a single probe were not reliable after resequencing. For a potential mSFP call, a conservative signal cutoff ratio of 3–5.0 and a vote among several adjacent probes (within 25 bp) with a majority of the probes (at least 75%) should be applied. We demonstrated that this particular microarray could be successfully employed to detect mSFP with high mSFP calling accuracy (≥ 94%). This work provides important information for calling mSFP in the *P. falicparum *genome using microarrays.

We used a 5.0 cutoff ratio in calling SFP because for genetic mapping, a high Fp rate may lead to misleading results that should be minimized. A higher cutoff value may result in a higher Fn rate or missing some calls too. Missing some calls will not be a big issue as the array can detect a large number of SFP. The 5.0 cutoff therefore represents a conservative value for minimizing Fp calls, considering potential higher backgrounds that may exist in some field isolates such as FCR3 in this study. Higher background in FCR3 requires further investigations, although signal intensity and distribution from this parasite appeared to be similar to those from other parasites (Additional file 1 and 2). A sample mixed with a smaller percentage of DNA from a different genotype (strain) may increase the hybridization background signal. Indeed, typing DNA from the FCR3 parasite with microsatellites showed that the DNA sample appeared to contain a secondary peak in some markers (data not shown). If this is true, a sample with high background may have to be discarded.

Using an array with a much higher density of probes than those published previously [27-29], we identified 121,087 mSFP from five isolates, including ~18,000 new mSFP after excluding mSFP from multigene families. Among the 121,087 mSFP, ~67% were in clusters of highly polymorphic genes such as *var/rif*/*stevor*. Approximately 89% of our mSFP calls that also had probes spanning known SNP in PlasmoDB matched the SNP, reflecting relative high accuracy of our mSFP calls, although our stringent cutoff values may lead to higher Fn rates or "no-calls" (such as excluding single probe calls). Our mSFP also provided additional evidence confirming the SNP reported previously, which is important because the majority of SNP in PlasmoDB were generated from shotgun sequences and sequence alignments have not been visually inspected or adjusted. For a genome with large number of repetitive sequences, sequence alignment errors can be generated if sequence alignment is totally relied on computer software [4].

Distributions of mSFP across the chromosomes among the parasites were very similar except for a few unique peaks that may reflect deletion or amplification in each individual parasite. If we exclude the mSFP from the multigene families, we obtained 40,354 mSFP or approximately 570 bp per SFP in the genome, a frequency that is within the range (519–976 bp per SNP) of our previous estimates [35] and similar to an estimate of 446 bp per SNP by another group [5]. If we consider 45% of the 40,354 mSFP from five isolates as common mSFP, as estimated previously [4], we can expect ~18,000 common mSFP in the five parasite genomes that will be useful for genetic mapping.

The highly AT-rich *P. falciparum *genome has a large number of repetitive sequences and low complexity regions in protein coding sequences [35-37]. The non-coding regions consist of more than 40% of the genome and generally have AT content >90% with large numbers of polymorphic AT repeats and polyA/T tracts [26,38]. These high-AT regions not only present a problem for genome sequencing and DNA sequence alignment but also make it difficult to design sequence-specific probes with reliable hybridization dynamics. SNP in these regions may not be very useful for mapping purposes because of difficulty in designing oligonucleotide probes or PCR primers for genotyping. Indeed, analyses of signal intensity from probes with different GC contents showed that probes with GC contents <16% produced similar low signals, suggesting that these probes might not be practical for calling mSFP. Of interest, probes with GC content >50% also produced highly variable signals. The majority of high-GC probes from the variable *var *genes can partly contribute to this variation. We excluded probes with GC content >50% for several reasons: 1) Approximately 44% of the probes with GC content >50% were *var *probes that should be discarded; 2) probes with high GC content would have higher 'affinity' than those with lower GC content during hybridization. A substitution in a probe with high GC content may not reduce the hybridization signal as much as a probe with low GC content; 3) there were only ~3000 probes with GC contents >50%. Exclusion of these probes should not have significant impact on our SFP calls.

The *P. falciparum *chromosomes have been shown to be highly variable in size in pulse-field gel electrophoresis (PFG) [39]. Genomic segmentation analysis to detect chromosome deletion and amplification showed relatively few amplification/deletion events with segment size > 0.3 kb. The variation in chromosome sizes seen in PFG gels could be mainly due to chromosome translocation, which is difficult if not impossible to detect using microarrays. One of the amplified regions was a segment on chromosome 5 containing the *pfmdr1 *gene in the Dd2 and FCR3 parasites. Amplification of the *pfmdr1 *locus has been reported [28,29,33], which could be due to drug selection pressure [40]. Similarly, there were few deletions larger than 10 kb; many of the deleted/amplified regions detected in our study matched well with those reported previously [28,29]. Two well-known deleted regions on chromosome 2 and 9, respectively, were detected in our analyses [34,41]. Detection of previously reported deletions suggested that our methods for detecting deletion/amplification were working properly. However, using an array with higher probe density than previous studies, we also discovered many deletions/amplifications that have not been described previously (Additional file 9). We identified 181 amplified and 536 highly variable or deleted genes or fragments, 74 (40.9%) and 30 (5.6%) of which, respectively, were reported previously [28,29,33]. Some of the discrepancies were likely due to different filtering criteria used (e.g. cutoff ratios, minimum number of probes, length cutoff of segment). Because of our small parasite sample size, it is difficult to make any functional inferences from the amplifications and deletions found in this study, although amplification at the *pfmdr1 *locus may be associated with responses to some anti-malarial drugs [40,42], and amplification of chromosome 4 in FCR3 may contribute to its adaptation to higher growth rates.

## Conclusion

This study developed methods for accurate detection of mSFP and CNV in the *P. falciparum *genome after evaluating factors that can influence DNA hybridization dynamics. More than 120,000 mSFP, including ~18,000 new and unique mSFP, and various chromosomal amplification/deletions were identified from the *P. falciparum *genome. Nearly 70% of the polymorphic sites are in clusters of *var/rif/stevor *gene families. Use of this array to analyze DNA samples from large numbers of parasites will facilitate our understanding of parasite diversity and evolution and genetic mapping of important parasite traits.

## Methods

### Parasites and parasite culture

*P. falciparum *parasite isolates used in this study have been described [4,43]. The parasites were cultured *in vitro *according to the methods of Trager and Jensen [44]. Briefly, parasites were maintained in RPMI 1640 medium containing 5% human O^+ ^erythrocytes (5% hematocrit), 0.5% Albumax (GIBCO, Life Technologies, Grand Island, NY), 24 mM sodium bicarbonate, and 10 μg/ml gentamycin at 37°C with 5% CO_2_, 5% O_2_, and 90% N_2_.

### DNA extraction and probe labeling

Parasites were cultured to a parasitemia of 5% or higher; and the cultures were centrifuged at 5000g to collect red blood cells that were lyzed with addition of 10 vol of 0.1% saponin in PBS. The parasites were centrifuged again; and genomic DNA was extracted from the parasite pellet using Wizard Genomic DNA Purification kit (Promega, Madison, WI). Genomic DNA (10 μg) from each parasite was used as probes in the hybridizations. Briefly, genomic DNA was fragmented to an average size of 50–150 bp with DNase I and the quality of the digested DNA evaluated in 2% agarose gels. Subsequently, fragmented DNA was end-labeled using terminal deoxynucleotidyl transferase and a biotin labeling kit (Affymetrix mapping 250 K reagent kit; Affymetrix, Inc., Santa Clara, CA).

### Microarray hybridization

The PFSANGER Genechip^® ^was purchased from Affymetrix, Inc. Array hybridization was performed at the microarray facility of the Laboratory of Immunopathogenesis and Bioinformatics, SAIC-Frederick, Inc (Frederick, MD). Briefly, biotin-labeled DNA were hybridized to array chips at 45°C for 16 h with constant rotation at 60 rpm. Affymetrix 20× hybridization control was used to make the hybridization cocktail. Hybridized chips were washed and stained following the company's EukGE-WS2v5 protocol. The chips were then scanned at 570 nm emission wavelength using an Affymetrix scanner 3000. All the parasites have two or more biological replicates (Additional file 1).

### Microarray chip design and data analysis

The probes were designed based on *P. falciparum *genome (3D7) sequence v2.1.1 [45] covering genomic regions where unique probes with a reasonably broad 'thermal' range could be designed. A brief description of the array design has been reported recently [46]. Because of recent updates of genome databases, all probe sequences were reassigned with new coordinates along each chromosome and their relative positions in a predicted gene (exon, intron, across exon and intron, and intergenic regions) according to the 3D7 genome sequence in PlasmoDB V5.2. The scanned image CEL files were processed and analyzed using the R/Bioconductor package and the robust multichip analysis method [47]. Basically, the programs retrieved probe information (perfect match only), performed background subtraction, quantile-normalized signals from the chips, and transformed the data into a final normalized data matrix of log2 values. Partek Genomics Suite 6.3 (Partek Inc., St. Louis, MO) and in-house programs are also used in SFP calling and copy number analyses.

### Mapping known SNP to array probes

After determining the correct genomic coordinates for each SNP and each array probe, known SNP from our previous study [4] and those in PlasmoDB [3,5,28,45] were mapped to probes that covered known SNP positions. Ambiguous SNP (mapped to multiple positions) were removed, and the remaining SNP were uploaded to a genome browser [32] with allele information from different parasites.

### SFP calling

Because the signals from the probes do not allow for accurate mapping of the position of a SNP within a probe at the given probe density, we can only assert that somewhere within a probe there is likely a polymorphism. Therefore, we simply assigned the polymorphism to a feature (probe) and called it a single feature polymorphism (SFP) as described [28]. Because a polymorphic site was often covered by multiple probes (average ~4 probes), we treated calls from probes within 25 bp as one SFP (called mSFP). To establish optimal parameters for SFP calling, we investigated SFP calling rates and calling accuracies using various conditions. We first identified all of the probes that covered each SNP identified in our previous study [4]. Then we extracted their hybridization signals from a normalized data file. The average probe intensity (average of antilogs of the raw data) from the normalized data for all replicates of each parasite isolate was calculated. This value was compared with the average signal for 3D7 obtained in the same way. A ratio was obtained after comparison with the signal from that of 3D7. We evaluated the influences of SNP position in a probe, GC content of a probe, cutoff ratios of hybridization signal, and numbers of probes on SFP calling accuracy. Probes with GC content < 16% and > 50%, and probes with multiple hits in the genome were excluded for the analyses. The last two nucleotides at each ends of a probe were also discarded, because substitutions at these positions had minimal influences on hybridization signals.

Once optimal parameters were identified for calling SNP using the NIAID SNP as an input set to test the method, we applied a similar procedure to a whole genome scan for probe-based SFP and mSFP (Additional file 9). Probe ratios were computed for each parasite for each probe, and raw alleles were generated by applying the cutoff ratio of 5.0 – it was an SFP if a ratio was above the cutoff value and it was not if below the ratio. Next, going through one parasite at a time, all probes were considered where there was more than one positive probe in a row within 25 bp of one another. Once this filtered set of probes was extracted from the full set, the ratios of intensity for each of the isolates compared with 3D7 was computed and tabulated. From this table, a vector was constructed for each parasite isolate where either a '1' or a '0' was added to each position determined by the value of the ratio. This vector was then scanned for stretches of '1's where the distance between the probes was less than 25 bp. In cases where longer stretches were identified, they were output as an additional feature type called long multiprobe polymorphism. Because some probes represent different strands of the exact same sequence region, we also discarded those stretches of '1's where the probes on either strand had a distance of 0 bp from the neighboring probe but did not exceed the threshold ratio value. All of the multiprobe polymorphisms corresponding to the mSFP were then output, and both classes of polymorphisms (single probe SFP and multi-probe mSFP) were then loaded into the genome browser. The procedure also tracks the 'alleles' by parasite isolate to determine the counts of mSFP shared by each possible combination of parasite isolates. Additional parameters that added confidence to a particular mSFP call, such as multiple parasite isolates having the same SFP and matches to known SNP in PlasmoDB, were also indicated.

### Estimating SFP calling rates using ROC curve and Z-score

Hybridization measurements from Affymetrix CEL files were pre-processed in the R programming environment [48] using the read.affybatch function from the affy BioConductor package [49]. Background adjustment was performed using the method developed for the RMA algorithm, and normalization was done using the quantile method. Differential hybridization between parasite isolates was expressed as Z-scores calculated by the LPE package [30,50].

### DNA sequencing

To verify selected mSNP (Table 2) that might be called incorrectly or calls that had contradictory signals, we amplified DNA fragments of 200–500 bp containing the probes and sequenced the PCR products directly according to methods described [43]. Primer sequences used in PCR and DNA sequencing are listed in Table 2.

### Detection of CNV

To detect CNV, we imported the filtered probe data into Partek Genomics Suite v6.3 and normalized individual probe signal from the 3D7 reference genome to 1.0 (haploid genome). Basically, the genomic segmentation algorithm finds a segment according to three criteria: 1) neighboring regions have statistically significantly different average intensities (*P *≥ 0.00001); 2) breakpoints (region boundaries) were chosen to give optimal statistical significance (smallest *P*-value); and 3) detected regions must contain a minimum of 15 probes. After determining the segments that had average signals higher or lower than 1.5 fold of those of the 3D7 reference, we filtered out regions that were less than 300 bp long. Detected segments, representing potential deletions or highly polymorphic regions, were plotted along chromosomes to produce CN genome view (Figure 5); and the segments were mapped to predicted genes in PlasmoDB to generate additional file 9. To screen for those highly polymorphic genes from potentially deleted segments, we flagged segments containing *var/rif/stevor *and other multigene families.

## Abbreviations

CNV: copy number variation; CV: coefficient of variance; Fn: false negative; Fp: false positive; LPE: local pooled error; MS: microsatellites; ROC: receiver operating characteristic; SFP: single feature polymorphism; mSFP: SFP called by two or more overlapping probes; SNP: single nucleotide polymorphism.

## Competing interests

The authors declare that they have no competing interests.

## Authors' contributions

HJ prepared parasite culture, performed DNA extraction, labeling, DNA sequencing, array hybridization, and data analysis, and took an active part in writing the manuscript; MY performed data analysis; JM and LZ performed parasite culture and MS typing as well as DNA extraction; AI and YH performed data analysis; LJK, performed ROC and z-score analyses; RMS performed data analysis and took an active part in writing the manuscript; X-zS designed the project, performed data analysis, and took an active part in writing the manuscript. All authors read and approved the manuscript.

## Supplementary Material

Additional file 1 Parasite sample replicates and basic hybridization statistics after normalization. Click here for file

Additional file 2 Plots of normalized signal ratios averaged from parasite replicates, showing distribution of probe signal ratios from each parasite (over 3D7). Click here for file

Additional file 3 Number of NIAID SNP that are covered by different numbers of probes. Click here for file

Additional file 4 The numbers of probes with NIAID SNP at positions 1–25. Probes with a single SNP are in light blue, two SNP are in red, and more than two SNP are in dark blue. Click here for file

Additional file 5 Summary of procedures for calling genome-wide SFP and copy number variation. Click here for file

Additional file 6 mSFP calls for the 14 chromosomes among five parasite isolates after excluding calls from multigene families. Click here for file

Additional file 7 Numbers of SFP, mSFP, and known SNP in predicted *P. falciparum *genes. Click here for file

Additional file 8 SFP counts per 10-kb bins across the 14 chromosomes from 7G8, Dd2, FCR3, and HB3. Click here for file

Additional file 9 Amplified and deleted chromosomal segments or genes 300 bp or larger. Click here for file

## References

[B1] Snow RW, Guerra CA, Noor AM, Myint HY, Hay SI (2005). The global distribution of clinical episodes of *Plasmodium falciparum *malaria. Nature.

[B2] Su X-z, Hayton K, Wellems TE (2007). Genetic linkage and association analyses for trait mapping in *Plasmodium falciparum*. Nat Rev Genet.

[B3] Jeffares DC, Pain A, Berry A, Cox AV, Stalker J, Ingle CE, Thomas A, Quail MA, Siebenthall K, Uhlemann AC, Kyes S, Krishna S, Newbold C, Dermitzakis ET, Berriman M (2007). Genome variation and evolution of the malaria parasite *Plasmodium falciparum*. Nat Genet.

[B4] Mu J, Awadalla P, Duan J, McGee KM, Keebler J, Seydel K, McVean GA, Su X-z (2007). Genome-wide variation and identification of vaccine targets in the *Plasmodium falciparum *genome. Nat Genet.

[B5] Volkman SK, Sabeti PC, DeCaprio D, Neafsey DE, Schaffner SF, Milner DA, Daily JP, Sarr O, Ndiaye D, Ndir O, Mboup S, Duraisingh MT, Lukens A, Derr A, Stange-Thomann N, Waggoner S, Onofrio R, Ziaugra L, Mauceli E, Gnerre S, Jaffe DB, Zainoun J, Wiegand RC, Birren BW, Hartl DL, Galagan JE, Lander ES, Wirth DF (2007). A genome-wide map of diversity in *Plasmodium falciparum*. Nat Genet.

[B6] Lindblad-Toh K, Winchester E, Daly MJ, Wang DG, Hirschhorn JN, Laviolette JP, Ardlie K, Reich DE, Robinson E, Sklar P, Shah N, Thomas D, Fan JB, Gingeras T, Warrington J, Patil N, Hudson TJ, Lander ES (2000). Large-scale discovery and genotyping of single-nucleotide polymorphisms in the mouse. Nat Genet.

[B7] Kennedy GC, Matsuzaki H, Dong S, Liu WM, Huang J, Liu G, Su X, Cao M, Chen W, Zhang J, Liu W, Yang G, Di X, Ryder T, He Z, Surti U, Phillips MS, Boyce-Jacino MT, Fodor SP, Jones KW (2003). Large-scale genotyping of complex DNA. Nat Biotechnol.

[B8] Gunderson KL, Steemers FJ, Lee G, Mendoza LG, Chee MS (2005). A genome-wide scalable SNP genotyping assay using microarray technology. Nat Genet.

[B9] Hardenbol P, Yu F, Belmont J, Mackenzie J, Bruckner C, Brundage T, Boudreau A, Chow S, Eberle J, Erbilgin A, Falkowski M, Fitzgerald R, Ghose S, Iartchouk O, Jain M, Karlin-Neumann G, Lu X, Miao X, Moore B, Moorhead M, Namsaraev E, Pasternak S, Prakash E, Tran K, Wang Z, Jones HB, Davis RW, Willis TD, Gibbs RA (2005). Highly multiplexed molecular inversion probe genotyping: over 10,000 targeted SNPs genotyped in a single tube assay. Genome Res.

[B10] Steemers FJ, Gunderson KL (2007). Whole genome genotyping technologies on the BeadArray platform. Biotechnol J.

[B11] Hagiwara H, Sawakami-Kobayashi K, Yamamoto M, Iwasaki S, Sugiura M, Abe H, Kunihiro-Ohashi S, Takase K, Yamane N, Kato K, Son R, Nakamura M, Segawa O, Yoshida M, Yohda M, Tajima H, Kobori M, Takahama Y, Itakura M, Machida M (2007). Development of an automated SNP analysis method using a paramagnetic beads handling robot. Biotechnol Bioeng.

[B12] Hakonarson H, Grant SF, Bradfield JP, Marchand L, Kim CE, Glessner JT, Grabs R, Casalunovo T, Taback SP, Frackelton EC, Lawson ML, Robinson LJ, Skraban R, Lu Y, Chiavacci RM, Stanley CA, Kirsch SE, Rappaport EF, Orange JS, Monos DS, Devoto M, Qu HQ, Polychronakos C (2007). A genome-wide association study identifies KIAA0350 as a type 1 diabetes gene. Nature.

[B13] Sladek R, Rocheleau G, Rung J, Dina C, Shen L, Serre D, Boutin P, Vincent D, Belisle A, Hadjadj S, Balkau B, Heude B, Charpentier G, Hudson TJ, Montpetit A, Pshezhetsky AV, Prentki M, Posner BI, Balding DJ, Meyre D, Polychronakos C, Froguel P (2007). A genome-wide association study identifies novel risk loci for type 2 diabetes. Nature.

[B14] Consortium WTCC (2007). Genome-wide association study of 14,000 cases of seven common diseases and 3,000 shared controls. Nature.

[B15] Scott LJ, Mohlke KL, Bonnycastle LL, Willer CJ, Li Y, Duren WL, Erdos MR, Stringham HM, Chines PS, Jackson AU, Prokunina-Olsson L, Ding CJ, Swift AJ, Narisu N, Hu T, Pruim R, Xiao R, Li XY, Conneely KN, Riebow NL, Sprau AG, Tong M, White PP, Hetrick KN, Barnhart MW, Bark CW, Goldstein JL, Watkins L, Xiang F, Saramies J, Buchanan TA, Watanabe RM, Valle TT, Kinnunen L, Abecasis GR, Pugh EW, Doheny KF, Bergman RN, Tuomilehto J, Collins FS, Boehnke M (2007). A genome-wide association study of type 2 diabetes in Finns detects multiple susceptibility variants. Science.

[B16] Saxena R, Voight BF, Lyssenko V, Burtt NP, de Bakker PI, Chen H, Roix JJ, Kathiresan S, Hirschhorn JN, Daly MJ, Hughes TE, Groop L, Altshuler D, Almgren P, Florez JC, Meyer J, Ardlie K, Bengtsson Bostrom K, Isomaa B, Lettre G, Lindblad U, Lyon HN, Melander O, Newton-Cheh C, Nilsson P, Orho-Melander M, Rastam L, Speliotes EK, Taskinen MR, Tuomi T, Guiducci C, Berglund A, Carlson J, Gianniny L, Hackett R, Hall L, Holmkvist J, Laurila E, Sjogren M, Sterner M, Surti A, Svensson M, Svensson M, Tewhey R, Blumenstiel B, Parkin M, Defelice M, Barry R, Brodeur W, Camarata J, Chia N, Fava M, Gibbons J, Handsaker B, Healy C, Nguyen K, Gates C, Sougnez C, Gage D, Nizzari M, Gabriel SB, Chirn GW, Ma Q, Parikh H, Richardson D, Ricke D, Purcell S (2007). Genome-wide association analysis identifies loci for type 2 diabetes and triglyceride levels. Science.

[B17] Winkelmann J, Schormair B, Lichtner P, Ripke S, Xiong L, Jalilzadeh S, Fulda S, Putz B, Eckstein G, Hauk S, Trenkwalder C, Zimprich A, Stiasny-Kolster K, Oertel W, Bachmann CG, Paulus W, Peglau I, Eisensehr I, Montplaisir J, Turecki G, Rouleau G, Gieger C, Illig T, Wichmann HE, Holsboer F, Muller-Myhsok B, Meitinger T (2007). Genome-wide association study of restless legs syndrome identifies common variants in three genomic regions. Nat Genet.

[B18] Buch S, Schafmayer C, Volzke H, Becker C, Franke A, von Eller-Eberstein H, Kluck C, Bassmann I, Brosch M, Lammert F, Miquel JF, Nervi F, Wittig M, Rosskopf D, Timm B, Holl C, Seeger M, Elsharawy A, Lu T, Egberts J, Fandrich F, Folsch UR, Krawczak M, Schreiber S, Nurnberg P, Tepel J, Hampe J (2007). A genome-wide association scan identifies the hepatic cholesterol transporter ABCG8 as a susceptibility factor for human gallstone disease. Nat Genet.

[B19] Tomlinson I, Webb E, Carvajal-Carmona L, Broderick P, Kemp Z, Spain S, Penegar S, Chandler I, Gorman M, Wood W, Barclay E, Lubbe S, Martin L, Sellick G, Jaeger E, Hubner R, Wild R, Rowan A, Fielding S, Howarth K, Silver A, Atkin W, Muir K, Logan R, Kerr D, Johnstone E, Sieber O, Gray R, Thomas H, Peto J, Cazier JB, Houlston R (2007). A genome-wide association scan of tag SNPs identifies a susceptibility variant for colorectal cancer at 8q24.21. Nat Genet.

[B20] Zanke BW, Greenwood CM, Rangrej J, Kustra R, Tenesa A, Farrington SM, Prendergast J, Olschwang S, Chiang T, Crowdy E, Ferretti V, Laflamme P, Sundararajan S, Roumy S, Olivier JF, Robidoux F, Sladek R, Montpetit A, Campbell P, Bezieau S, O'Shea AM, Zogopoulos G, Cotterchio M, Newcomb P, McLaughlin J, Younghusband B, Green R, Green J, Porteous ME, Campbell H, Blanche H, Sahbatou M, Tubacher E, Bonaiti-Pellie C, Buecher B, Riboli E, Kury S, Chanock SJ, Potter J, Thomas G, Gallinger S, Hudson TJ, Dunlop MG (2007). Genome-wide association scan identifies a colorectal cancer susceptibility locus on chromosome 8q24. Nat Genet.

[B21] Bignell GR, Huang J, Greshock J, Watt S, Butler A, West S, Grigorova M, Jones KW, Wei W, Stratton MR, Futreal PA, Weber B, Shapero MH, Wooster R (2004). High-resolution analysis of DNA copy number using oligonucleotide microarrays. Genome Res.

[B22] Huang J, Wei W, Chen J, Zhang J, Liu G, Di X, Mei R, Ishikawa S, Aburatani H, Jones KW, Shapero MH (2006). CARAT: a novel method for allelic detection of DNA copy number changes using high density oligonucleotide arrays. BMC Bioinformatics.

[B23] Komura D, Shen F, Ishikawa S, Fitch KR, Chen W, Zhang J, Liu G, Ihara S, Nakamura H, Hurles ME, Lee C, Scherer SW, Jones KW, Shapero MH, Huang J, Aburatani H (2006). Genome-wide detection of human copy number variations using high-density DNA oligonucleotide arrays. Genome Res.

[B24] Hua J, Craig DW, Brun M, Webster J, Zismann V, Tembe W, Joshipura K, Huentelman MJ, Dougherty ER, Stephan DA (2007). SNiPer-HD: improved genotype calling accuracy by an expectation-maximization algorithm for high-density SNP arrays. Bioinformatics.

[B25] Carvalho B, Bengtsson H, Speed TP, Irizarry RA (2007). Exploration, normalization, and genotype calls of high-density oligonucleotide SNP array data. Biostatistics.

[B26] Gardner MJ, Hall N, Fung E, White O, Berriman M, Hyman RW, Carlton JM, Pain A, Nelson KE, Bowman S, Paulsen IT, James K, Eisen JA, Rutherford K, Salzberg SL, Craig A, Kyes S, Chan MS, Nene V, Shallom SJ, Suh B, Peterson J, Angiuoli S, Pertea M, Allen J, Selengut J, Haft D, Mather MW, Vaidya AB, Martin DM, Fairlamb AH, Fraunholz MJ, Roos DS, Ralph SA, McFadden GI, Cummings LM, Subramanian GM, Mungall C, Venter JC, Carucci DJ, Hoffman SL, Newbold C, Davis RW, Fraser CM, Barrell B (2002). Genome sequence of the human malaria parasite *Plasmodium falciparum*. Nature.

[B27] Carret CK, Horrocks P, Konfortov B, Winzeler E, Qureshi M, Newbold C, Ivens A (2005). Microarray-based comparative genomic analyses of the human malaria parasite *Plasmodium falciparum *using Affymetrix arrays. Mol Biochem Parasitol.

[B28] Kidgell C, Volkman SK, Daily J, Borevitz JO, Plouffe D, Zhou Y, Johnson JR, Le Roch K, Sarr O, Ndir O, Mboup S, Batalov S, Wirth DF, Winzeler EA (2006). A systematic map of genetic variation in *Plasmodium falciparum*. PLoS Pathog.

[B29] Ribacke U, Mok BW, Wirta V, Normark J, Lundeberg J, Kironde F, Egwang TG, Nilsson P, Wahlgren M (2007). Genome wide gene amplifications and deletions in *Plasmodium falciparum*. Mol Biochem Parasitol.

[B30] Jain N, Thatte J, Braciale T, Ley K, O'Connell M, Lee JK (2003). Local-pooled-error test for identifying differentially expressed genes with a small number of replicated microarrays. Bioinformatics.

[B31] Siewert B, Bly BM, Schlaug G, Darby DG, Thangaraj V, Warach S, Edelman RR (1996). Comparison of the BOLD- and EPISTAR-technique for functional brain imaging by using signal detection theory. Magn Reson Med.

[B32] ABCC GB. http://p-falcip.abcc.ncifcrf.gov/cgi-bin/gbrowse/malaria_sanger.

[B33] Triglia T, Foote SJ, Kemp DJ, Cowman AF (1991). Amplification of the multidrug resistance gene *pfmdr1 *in *Plasmodium falciparum *has arisen as multiple independent events. Mol Cell Biol.

[B34] Day KP, Karamalis F, Thompson J, Barnes DA, Peterson C, Brown H, Brown GV, Kemp DJ (1993). Genes necessary for expression of a virulence determinant and for transmission of *Plasmodium falciparum *are located on a 0.3-megabase region of chromosome 9. Proc Natl Acad Sci USA.

[B35] Mu J, Duan J, Makova K, Joy DA, Huynh CQ, Branch OH, Li W-h, Su X-z (2002). Chromosome-wide SNPs reveal an ancient origin for *Plasmodium falciparum*. Nature.

[B36] Pizzi E, Frontali C (2001). Low-complexity regions in *Plasmodium falciparum *proteins. Genome Res.

[B37] Aravind L, Iyer LM, Wellems TE, Miller LH (2003). Plasmodium biology: genomic gleanings. Cell.

[B38] Su X-z, Wellems TE (1996). Toward a high-resolution *Plasmodium falciparum *linkage map: polymorphic markers from hundreds of simple sequence repeats. Genomics.

[B39] Kemp DJ, Corcoran LM, Coppel RL, Stahl HD, Bianco AE, Brown GV, Anders RF (1985). Size variation in chromosomes from independent cultured isolates of *Plasmodium falciparum*. Nature.

[B40] Price RN, Uhlemann AC, Brockman A, McGready R, Ashley E, Phaipun L, Patel R, Laing K, Looareesuwan S, White NJ, Nosten F, Krishna S (2004). Mefloquine resistance in *Plasmodium falciparum *and increased *pfmdr1 *gene copy number. Lancet.

[B41] Biggs BA, Kemp DJ, Brown GV (1989). Subtelomeric chromosome deletions in field isolates of *Plasmodium falciparum *and their relationship to loss of cytoadherence i*n vitro*. Proc Natl Acad Sci USA.

[B42] Cowman AF, Galatis D, Thompson JK (1994). Selection for mefloquine resistance in *Plasmodium falciparum *is linked to amplification of the *pfmdr1 *gene and cross-resistance to halofantrine and quinine. Proc Natl Acad Sci USA.

[B43] Mu J, Ferdig MT, Feng X, Joy DA, Duan J, Furuya T, Subramanian G, Aravind L, Cooper RA, Wootton JC, Xiong M, Su X-z (2003). Multiple transporters associated with malaria parasite responses to chloroquine and quinine. Mol Microbiol.

[B44] Trager W, Jensen JB (1976). Human malaria parasites in continuous culture. Science.

[B45] Plasmo DB *Plasmodium falciparum *genome 3D7 sequence v2.1.1. http://www.plasmodb.org/plasmo/home.jsp.

[B46] Mourier T, Carret C, Kyes S, Christodoulou Z, Gardner PP, Jeffares DC, Pinches R, Barrell B, Berriman M, Griffiths-Jones S, Ivens A, Newbold C, Pain A (2008). Genome-wide discovery and verification of novel structured RNAs in *Plasmodium falciparum*. Genome Res.

[B47] Irizarry K, Kustanovich V, Li C, Brown N, Nelson S, Wong W, Lee CJ (2000). Genome-wide analysis of single-nucleotide polymorphisms in human expressed sequences. Nat Genet.

[B48] Team RDC (2006). R: A language and environment for statistical computing.

[B49] Gautier L, Cope L, Bolstad BM, Irizarry RA (2004). affy – analysis of Affymetrix GeneChip data at the probe level. Bioinformatics.

[B50] R, package version 1.8.0. http://www.r-project.org.

