# Supplementary material for: Detection of genome-wide polymorphisms in the AT-rich Plasmodium falciparum genome using a high-density microarray
Source: BMC Genomics. 2008 Aug 25;9:398. doi: 10.1186/1471-2164-9-398 (PMC2543026; doi:10.1186/1471-2164-9-398)
Supplement: Additional file 1 — Parasite sample replicates and basic hybridization statistics after normalization. [file 1471-2164-9-398-S1.doc]

**Additional file** **1.** Parasite sample replicates and basic hybridization

statistics after normalization.

Isolates Origin # Repeat CV<25% Median(lg2) STD

3D7 Europe 4 91.10% 8.24 0.36

7G8 Brazil 3 94.90% 8.23 0.24

Dd2 Indochina 3 92.4% 8.23 0.29

FCR3 S. Asia 2 93.6% 8.21 0.25

HB3 Honduras 3 95.2% 8.24 0.29
