# Supplementary figures and images for: Detection of genome-wide polymorphisms in the AT-rich Plasmodium falciparum genome using a high-density microarray
Source: BMC Genomics. 2008 Aug 25;9:398. doi: 10.1186/1471-2164-9-398 (PMC2543026; doi:10.1186/1471-2164-9-398)

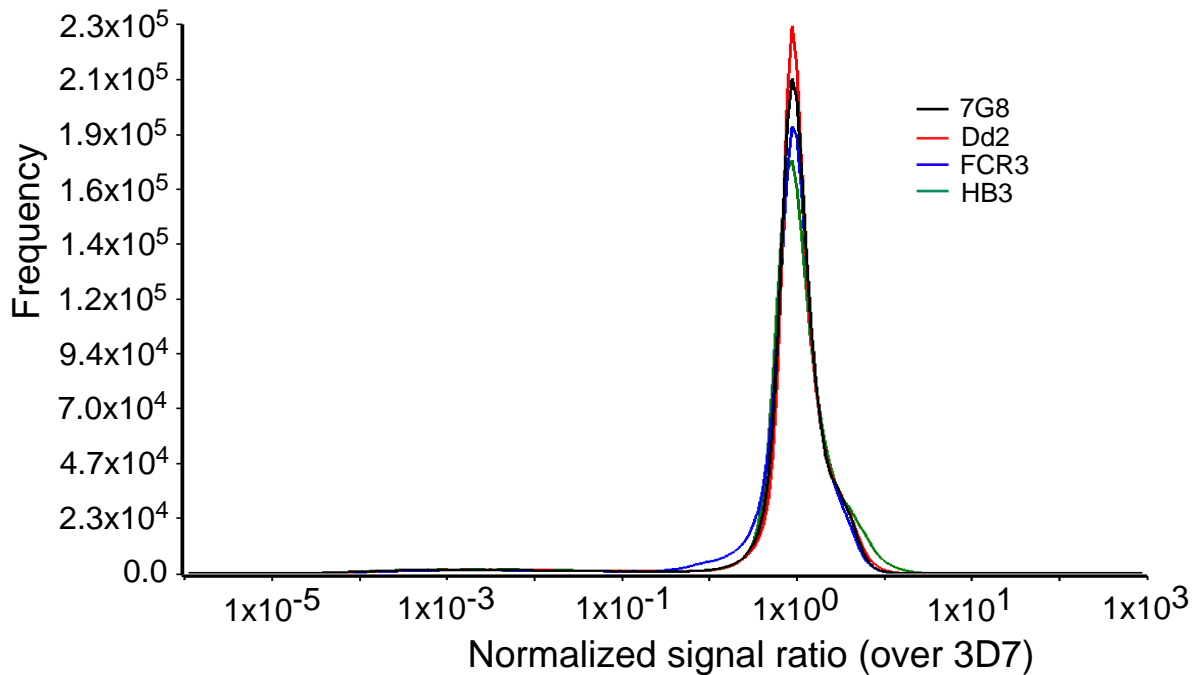

Supplement: Additional file 2 — Plots of normalized signal ratios averaged from parasite replicates, showing distribution of probe signal ratios from each parasite (over 3D7). [file 1471-2164-9-398-S2.pdf]

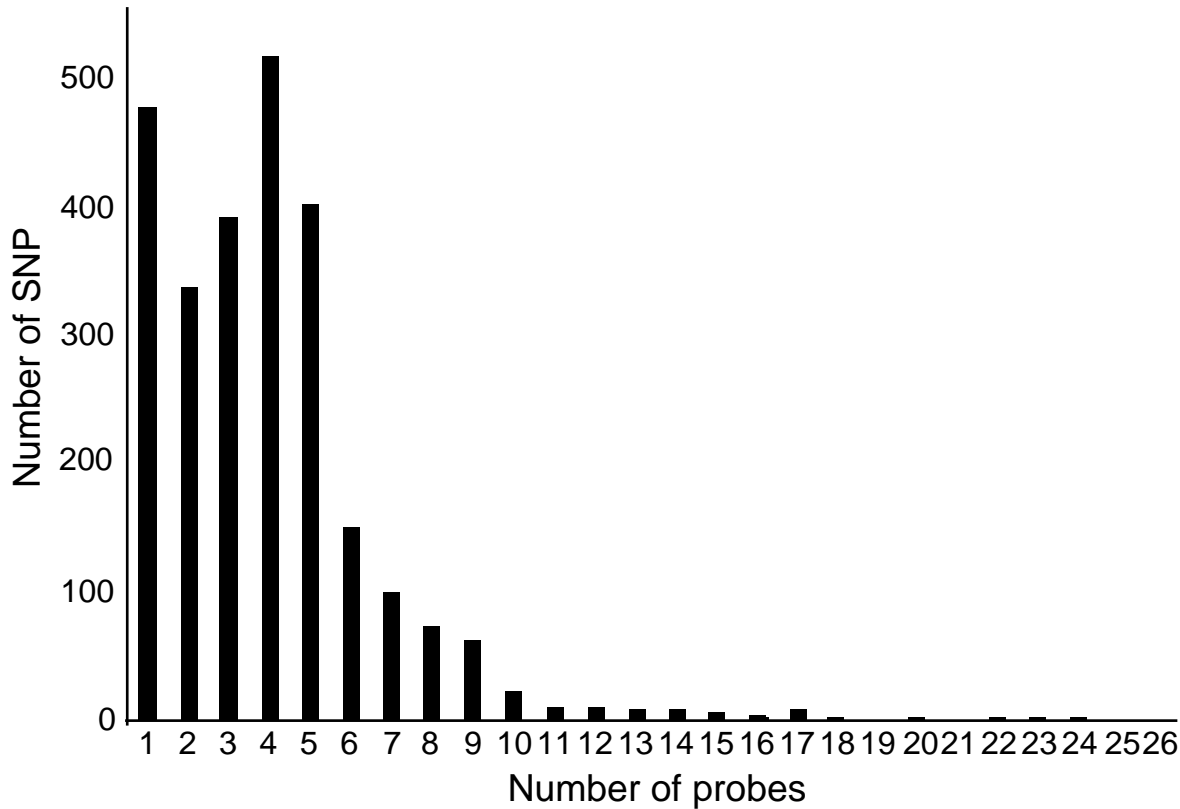

Supplement: Additional file 3 — Number of NIAID SNP that are covered by different numbers of probes. [file 1471-2164-9-398-S3.pdf]

Number of probes

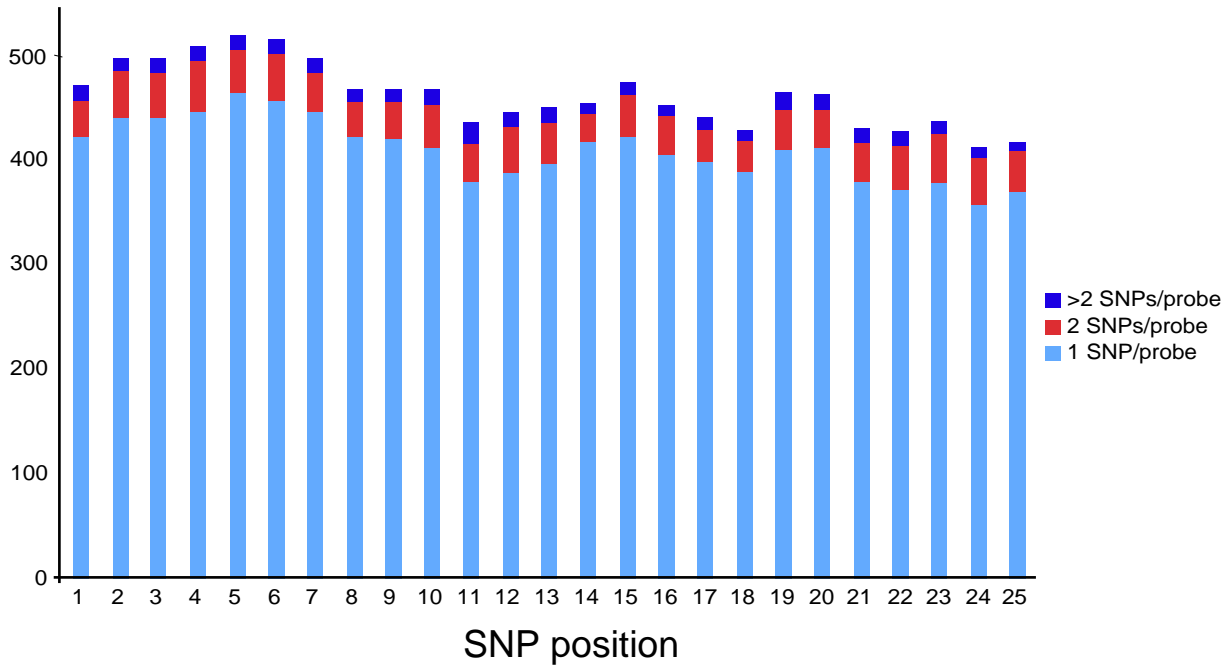

Supplement: Additional file 4 — The numbers of probes with NIAID SNP at positions 1–25. Probes with a single SNP are in light blue, two SNP are in red, and more than two SNP are in dark blue. [file 1471-2164-9-398-S4.pdf]

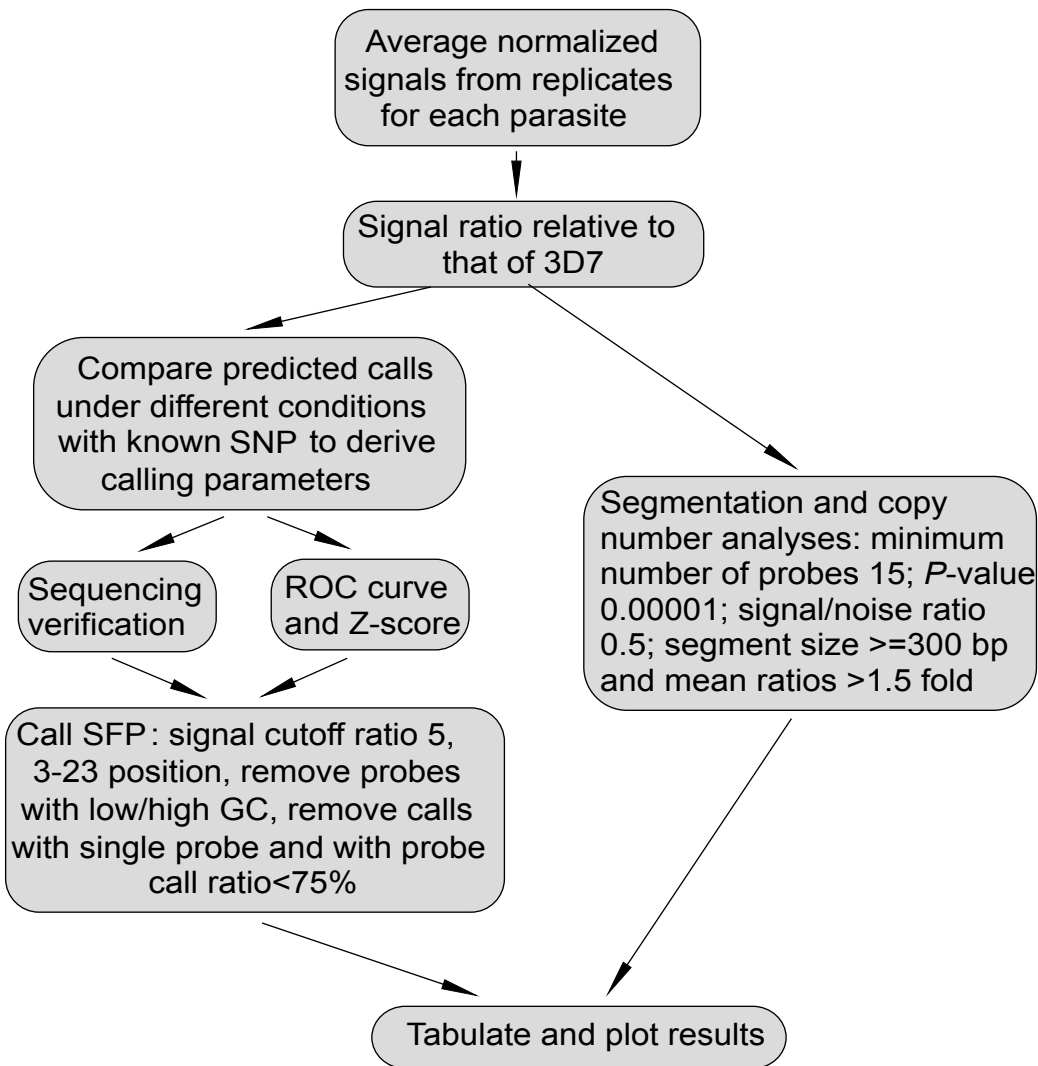

Supplement: Additional file 5 — Summary of procedures for calling genome-wide SFP and copy number variation. [file 1471-2164-9-398-S5.pdf]
