# Supplementary material for: Detection of genome-wide polymorphisms in the AT-rich Plasmodium falciparum genome using a high-density microarray
Source: BMC Genomics. 2008 Aug 25;9:398. doi: 10.1186/1471-2164-9-398 (PMC2543026; doi:10.1186/1471-2164-9-398)
Supplement: Additional file 6 — mSFP calls for the 14 chromosomes among five parasite isolates after excluding calls from multigene families. [file 1471-2164-9-398-S6.doc]

**Additional file 6.** mSFP calls for the 14 chromosomes among five parasite isolates after excluding calls from multigene families

| Isolate | Ch1 | Ch2 | Ch3 | Ch4 | Ch5 | Ch6 | Ch7 | Ch8 | Ch9 | Ch10 | Ch11 | Ch12 | Ch13 | Ch14 | Total |
| --- | --- | --- | --- | --- | --- | --- | --- | --- | --- | --- | --- | --- | --- | --- | --- |
| 0000* | 30311 | 50667 | 65655 | 58950 | 88790 | 84116 | 82429 | 84473 | 96421 | 186196 | 220779 | 205361 | 337017 | 227062 | 1818227 |
| 0001 | 70 | 370 | 314 | 253 | 350 | 394 | 373 | 284 | 557 | 551 | 482 | 693 | 1132 | 1032 | 6855 |
| 0010 | 38 | 47 | 90 | 119 | 77 | 48 | 85 | 111 | 1112 | 313 | 249 | 123 | 246 | 196 | 2854 |
| 0011 | 11 | 7 | 7 | 73 | 14 | 3 | 12 | 29 | 82 | 20 | 48 | 45 | 60 | 43 | 454 |
| 0100 | 117 | 1386 | 285 | 325 | 292 | 280 | 455 | 422 | 406 | 538 | 614 | 568 | 802 | 683 | 7173 |
| 0101 | 5 | 66 | 31 | 92 | 51 | 29 | 45 | 35 | 38 | 77 | 67 | 67 | 65 | 87 | 755 |
| 0110 | 43 | 108 | 139 | 125 | 98 | 133 | 166 | 97 | 136 | 212 | 193 | 216 | 370 | 200 | 2236 |
| 0111 | 7 | 49 | 58 | 57 | 102 | 62 | 80 | 90 | 82 | 96 | 107 | 138 | 251 | 186 | 1365 |
| 1000 | 140 | 205 | 288 | 348 | 309 | 326 | 346 | 374 | 362 | 730 | 526 | 487 | 1117 | 784 | 6342 |
| 1001 | 25 | 60 | 53 | 79 | 75 | 87 | 161 | 94 | 126 | 257 | 167 | 151 | 265 | 179 | 1779 |
| 1010 | 1 | 22 | 25 | 50 | 35 | 33 | 14 | 30 | 16 | 71 | 49 | 16 | 41 | 23 | 426 |
| 1011 | 7 | 30 | 8 | 27 | 25 | 25 | 13 | 12 | 60 | 15 | 32 | 60 | 64 | 57 | 435 |
| 1100 | 18 | 47 | 33 | 73 | 52 | 49 | 65 | 24 | 47 | 74 | 70 | 58 | 98 | 90 | 798 |
| 1101 | 14 | 63 | 53 | 81 | 52 | 22 | 77 | 44 | 60 | 92 | 91 | 136 | 87 | 51 | 923 |
| 1110 | 26 | 71 | 62 | 66 | 89 | 44 | 107 | 118 | 48 | 63 | 154 | 163 | 216 | 114 | 1341 |
| 1111 | 96 | 337 | 297 | 321 | 260 | 297 | 304 | 376 | 370 | 580 | 606 | 677 | 1232 | 865 | 6618 |
| Total | 618 | 2868 | 1743 | 2089 | 1881 | 1832 | 2303 | 2140 | 3502 | 3689 | 3455 | 3598 | 6046 | 4590 | 40354 |

*Parasite isolate order is 7G8, Dd2, FCR3, and HB3. For example, ‘1000’ indicates the numbers of unique alleles for 7G8. A ‘0’ indicates that a parasite has the same allele as that of 3D7 (0), and ‘1’ indicates a different allele (a mSFP). The numbers in the first row were positions with probes but no SFP were called. These numbers were not counted in the total calculation. The counts were based on a signal cutoff value of 5.0. Note that SFP from overlapping probes within 25 bp (or probe sets) were called as one SFP (mSFP).
